# No evident causal association between *Helicobacter pylori* infection and colorectal cancer: a bidirectional mendelian randomization study

**DOI:** 10.1038/s41598-023-45545-x

**Published:** 2023-10-29

**Authors:** Fang Luo, Peipei Zhou, Xiong Ran, Ming Gu, Shaoquan Zhou

**Affiliations:** 1 Department of Gastroenterology, Chongqing Hospital of Traditional Chinese Medicine, Chongqing, 400021 China; 2 Department of Radiology, Chongqing General Hospital, No.118, Xingguang Avenue, Liangjiang New District, Chongqing, 400014 China

**Keywords:** Cancer, Cancer genetics

## Abstract

Observational studies have reported a correlation between *Helicobacter pylori* infection and colorectal cancer (CRC); however, the underlying cause has remained unclear. This research was aimed at determining whether there is a correlation between *H. pylori* infection and CRC by measuring the prevalence of *H. pylori* CagA antibodies and VacA antibodies. Using data from many genome-wide association studies (GWAS), we conducted a Mendelian randomization (MR) study with two sample GWAS. Then, we used bidirectional MR to evaluate the association between *H. pylori* infection and CRC for identifying causation. The most common method of analysis was the inverse variance-weighted technique. In addition, we performed supplementary analyses using the weighted median technique and MR-Egger regression. Horizontal pleiotropic outliers were identified and corrected using the MR Pleiotropy RESidual Sum and Outlier (MR-PRESSO) method. Genetically predicted anti-*H. pylori* IgG seropositivity was not causally associated with CRC [odds ratio (OR): 1.12; 95% confidence interval (CI): 0.98–1.27, *P* = 0.08] and neither were *H. pylori* VacA antibody levels (OR = 0.96, 95% CI: 0.90–1.02, *P* = 0.25) or *H. pylori* CagA antibody levels (OR = 1.00, 95% CI: 0.93–1.07, *P* = 0.92). Furthermore, reverse MR analysis did not reveal evidence for a causal effect of CRC on *H. pylori* infection. The weighted median, the MR-Egger method, and MR-PRESSO yielded identical results. Using genetic data, MR analysis showed there was no evidence for a causal association between seroprevalence of *H. pylori* infection and CRC. The relationship between *H. pylori* infection and CRC requires further research.

## Introduction

Colorectal cancer (CRC) is the third most common malignancy worldwide and the second greatest cause of cancer-related mortality. It has the highest occurrence rate of all malignancies in individuals aged under 50 years^1^. Approximately 1.9 million newly diagnosed cases of CRC were reported in 2020, accounting for 10% of all malignancies worldwide^2^. The prevalence of CRC causes serious economic and societal burdens. Available data on its etiology suggest that its prevalence is influenced by advanced age, sex, ethnicity, family history of colorectal cancer, familial polyposis syndrome, chronic inflammatory bowel diseases, physical inactivity, unhealthy diet, geographical variation, and consumption of alcohol and cigarettes^3^.

*Helicobacter pylori* is a gram-negative bacteria that colonizes the stomach mucosa of humans and causes gastritis in around half the world’s population^4^. The primary virulence pathogenic factors of *H. pylori* infection are vacuolar cytotoxin A (VacA) and cytotoxin-associated protein A (CagA). Gastritis, duodenal ulcers, gastric cancer, and other GI and non-GI problems have all been found to be associated with *H. pylori* infection^5^. Epidemiological studies have recently focused on the association between *H. pylori* infection and CRC^6,7^. There is also evidence that a high incidence of *H. pylori* seropositivity in patients with CRC^8,9^ despite several reports stating that *H. pylori* infection has no role in the genesis of CRC^10,11^. Furthermore, several studies have even confirmed the presence of *H. pylori* in CRC or colonic polyps^12,13^. Notably, the evidence available so far is based on observational research. Chronic inflammation plays a role in the development of both H. pylori-associated diseases, such as gastritis, and CRC. Inflammatory conditions, such as inflammatory bowel disease (IBD), can confound the genetic associations by influencing both H. pylori infection status and CRC risk^14^. It is difficult to conduct a randomized controlled trial to check for an association between *H. pylori* infection and CRC. Consequently, elucidating if *H. pylori* infection is associated with CRC is challenging. Owing to the high prevalence of *H. pylori* infections in the population and the relative ease and cost-effectiveness of anti-*H. pylori* therapy^15^, identifying any association between *H. pylori* infection and CRC risk is of great public health and clinical importance.

To quantify the association of single nucleotide polymorphisms (SNPs) and *H. pylori* infection *with* CRC risk, we used a Mendelian randomization (MR) technique^16^ based on genetic variance index exposures to establish causal relationships of disease-related risk factors. Using descriptive data from large-scale genome-wide association studies (GWAS) of *H. pylori* infection and CRC, we used two-sample MR to test the hypothesis that *H. pylori* infection is associated with increased risk of developing CRC.

## Materials and methods

### Mendelian randomization design

**Figure 1:**
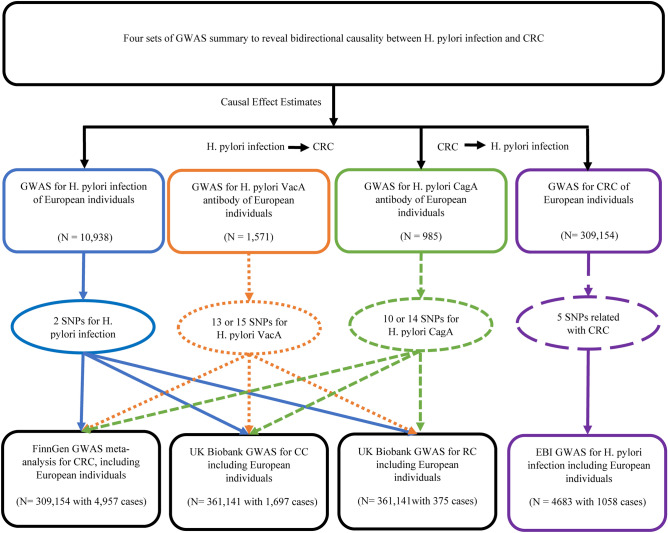
The workflow of the bidirectional MR study on the causal relationship between *H. pylori* infection and CRC.

Figure 1 shows the overall setup for the current MR study. Large-scale GWAS summary data are now more readily available, making MR a valuable tool in epidemiological investigations^17^. As shown in Supplementary Fig. S1, three crucial instrumental variable (IV) assumptions were used as the foundation for the MR study’s assumed validity: (a) relevance assumption: genetic variations are assumed to be highly correlated with exposure; (b) independence assumption: there are no unobserved confounders associated with the genetic variants; and (c) exclusion restriction: genetic polymorphisms are not linked to the outcome when exposure and other confounding factors are present^18^. To further evaluate the efficacy of the IVs, the F-statistic was calculated for each SNP using the following formula:

$$F={R}^{2}*\frac{\left(N-2\right)}{1-{R}^{2}},$$

where in R^2^ is the amount of exposure variation that can be explained by each genetic variant and N is the number of people in each sample^19^. An F-statistic of < 10 indicated that weak IVs may have skewed the direction of the confounded, observational relationship between phenotype and outcome^20^.

### GWAS summary data

Genetic associations with CRC were acquired from FinnGen consortium (https://www.finngen.fi/en) data version 7 (R7 release version 1 June 2022); this GWAS included 4957 CRC cases and 304,197 controls. The GWAS analyses were adjusted in the FinnGen study in terms of sex, major genetic components, and the genotyping batch^21^. To further elucidate the presence of a causal association between *H. pylori* infection and CRC, two GWAS studies were used to derive genetic associations for colon cancer (CC) and rectal cancer (RC), and both these studies used data from the UK Biobank study (http://www.nealelab.is/uk-biobank/); one of these studies comprised 1697 cases and 359,444 controls and the other comprised 375 cases and 360,766 controls. Pooled GWAS data from anti-*H. pylori* IgG levels were determined from public data compiled in the EBI database^22^ (https://gwas.mrcieu.ac.uk/datasets/ieu-b-4905/), covering 1058 European cases and 3,625 European controls, as shown in Table 1.

**Table 1:** Details of the studies in the mendelian randomization analysis.

Exposure/outcome	Consortium	Population	Sample size	URL
Anti-*H. pylori* IgG levels	EBI	European	4683	https://gwas.mrcieu.ac.uk/datasets/ieu-b-4905/
*H. pylori* VacA antibody levels	EBI	European	1571	https://gwas.mrcieu.ac.uk/datasets/ebi-a-GCST90006916/
*H. pylori* CagA antibody levels	EBI	European	985	https://gwas.mrcieu.ac.uk/datasets/ebi-a-GCST90006911/
RC	UK Biobank	European	361,141	http://www.nealelab.is/uk-biobank
CC	UK Biobank	European	361,141	http://www.nealelab.is/uk-biobank
CRC	FinnGen (R7)	European	309,154	https://www.finngen.fi/en

*H. pylori* *Helicobacter pylori*, *CRC* colorectal cancer, *EBI* European Bioinformatics Institute.

### Selection of the genetic instruments

Genetic IVs may be obtained via either a literature search or a GWAS summary data analysis. The Rotterdam studies RS-I, RS-II, and RS-III all yielded *H. pylori*-seropositive genetic IVs. Mayerle et al.^23^ performed a study on a European population that included 2763 cases and 8175 controls. As shown in Table 2, two SNPs (rs10004195 and rs368433) were identified to be independently and strongly associated with *H. pylori* seropositivity and have therefore been labeled as IVs. SNPs associated with anti-*H. pylori* VacA from the GWAS meta-analysis of EBI data (https://gwas.mrcieu.ac.uk/datasets/ebi-a-GCST90006916/) included 1,571 samples with 9,178,635 SNPs, of which 13 SNPs with *P* values < 5 × 10^–6^ were considered to be associated with VacA and used as IVs. Similarly, anti-*H. pylori* CagA from the EBI database (https://gwas.mrcieu.ac.uk/datasets/ebi-a-GCST90006911/) included 985 samples with 9,165,056 SNPs, of which ten SNPs with *P* values < 5 × 10^–6^ were considered to be associated with CagA and chosen as IVs.

**Table 2:** Instrument variants of *H. pylori* infection and F-statistic.

SNPs	Beta	SE	EAF	*P* value	Gene	R^2^ (%)	F-statistic
rs10004195A	−0.36	0.04	0.25	1.40E−18	TLR1	4.77	547.85
rs368433C	0.31	0.06	0.16	2.10E−08	FCGR2A	2.66	299.10

*SNPs* single-nucleotide polymorphisms, *EAF* effect allele frequency.

To verify reverse causality between *H. pylori* infection and CRC risk. we obtained genetic IVs of CRC from the FinnGen study, and *H. pylori* IgG seropositivity from the EBI database (https://gwas.mrcieu.ac.uk/datasets/ieu-b-4905/) as the outcome for reverse causal inference. First, to search for the CRC genetic IVs that met the assumptions of MR relevance, the SNPs had to reach genome-wide significance with a *P* value of 5 × 10^–8^. Second, we then checked for linkage disequilibrium (r^2^ < 0.001 and clump distance > 10,000 kb). Third, when we harmonized the exposure and outcome datasets, we eliminated SNPs with intermediate allele frequencies and ambiguous SNPs with mismatched alleles to guarantee that effect alleles belonged to the same allele. Fourth, to avoid the effect of weak IVs on the causal effect, the F-statistic of each selected IV had to be greater than 10. Finally, PhenoScanner V2 (http://www.phenoscanner.medschl.cam.ac.uk/) was used to test the hypothesis that IVs are independent of confounders and outcomes by analyzing genome-wide significant relationships (*P* < 5 × 10^–8^). After the above criteria were satisfied, five SNPs associated with CRC were selected as IVs for subsequent analysis, as shown in Supplementary Table S1.

### Ethics statement

This MR study was conducted using GWAS summary statistics or shared datasets. All of these data were anonymized, freely downloadable, and could be used without restriction. Therefore, a separate ethics statement or consent was not required. The study was approved by the hospital’s Institutional Review Board (Ethics 28 Committee of Chongqing General Hospital, ID: XJS S2022-052-01).

### Statistical analyses

Using a two-sample Mendelian randomization (MR) approach, we determined the cause of *H. pylori* infection and its reverse causality with CRC. The inverse variance-weighted (IVW) technique has been used as the main statistical model^24^. We estimated genetic predictive associations for seropositive *H. pylori* using the IVW fixed-effects method as only two SNPs were available^25^. The weighted median^26^ and MR-Egger^27^ methods were used for the sensitivity analyses of MR analyses involving more than two IVs. After removing potentially misleading outliers, we used the MR-PRESSO test to analyze and compensate for horizontal pleiotropy and provide causal estimates^28^. Cochrane Q values were used to evaluate heterogeneity among the estimated s of SNPs in each analysis, with a Cochran Q-derived *P* value of < 0.05 indicating possible horizontal pleiotropy^29^. The results of multiple comparisons were recalculated using Bonferroni's procedure^30^, and a *P* value of < 0.005 (0.05/10) was considered to indicate a causal connection. Using the mRnd website (https://shiny.cnsgenomics.com/mRnd/), we determined the statistical power of the experiment^31^. All analyses were two-sided and implemented in R (version 4.2.1) through the TwoSampleMR package (version 0.5.6). Forest plots were created using the “forestplot” R package (version 3.1.1).

## Results

### Causal effect of *H. pylori* infection and subtypes on CRC

It was discovered that the two SNPs known as the IVs rs10004195A and rs368433C were strongly and independently associated with *H. pylori* seropositivity. The F-statistic for these IVs was 547.85 and 299.10, respectively, thus avoiding the impact of weak IVs on the causal effect.

**Figure 2:**
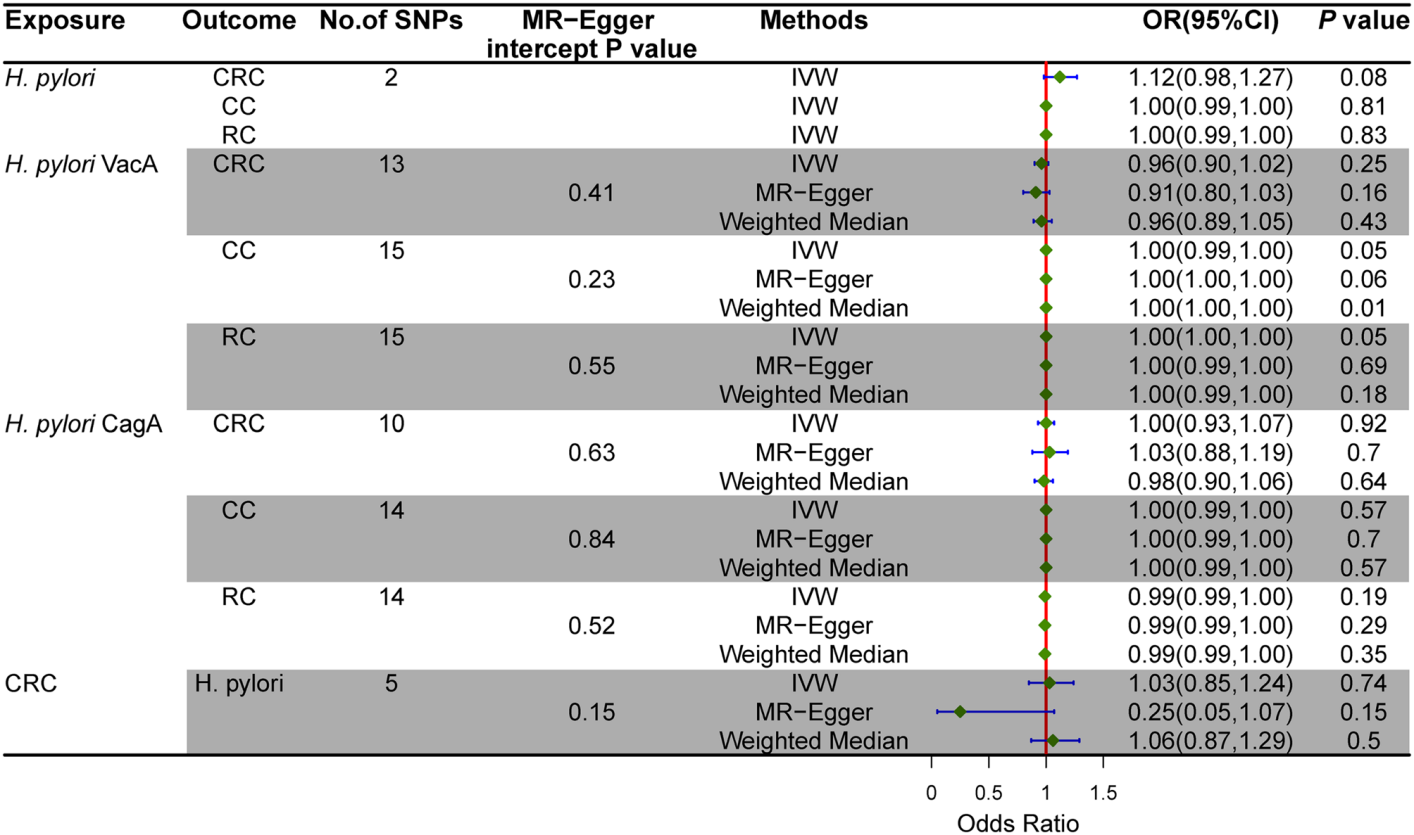
Estimated causal effects between *H. pylori* infection and CRC using different MR methods.

**Figure 3:**
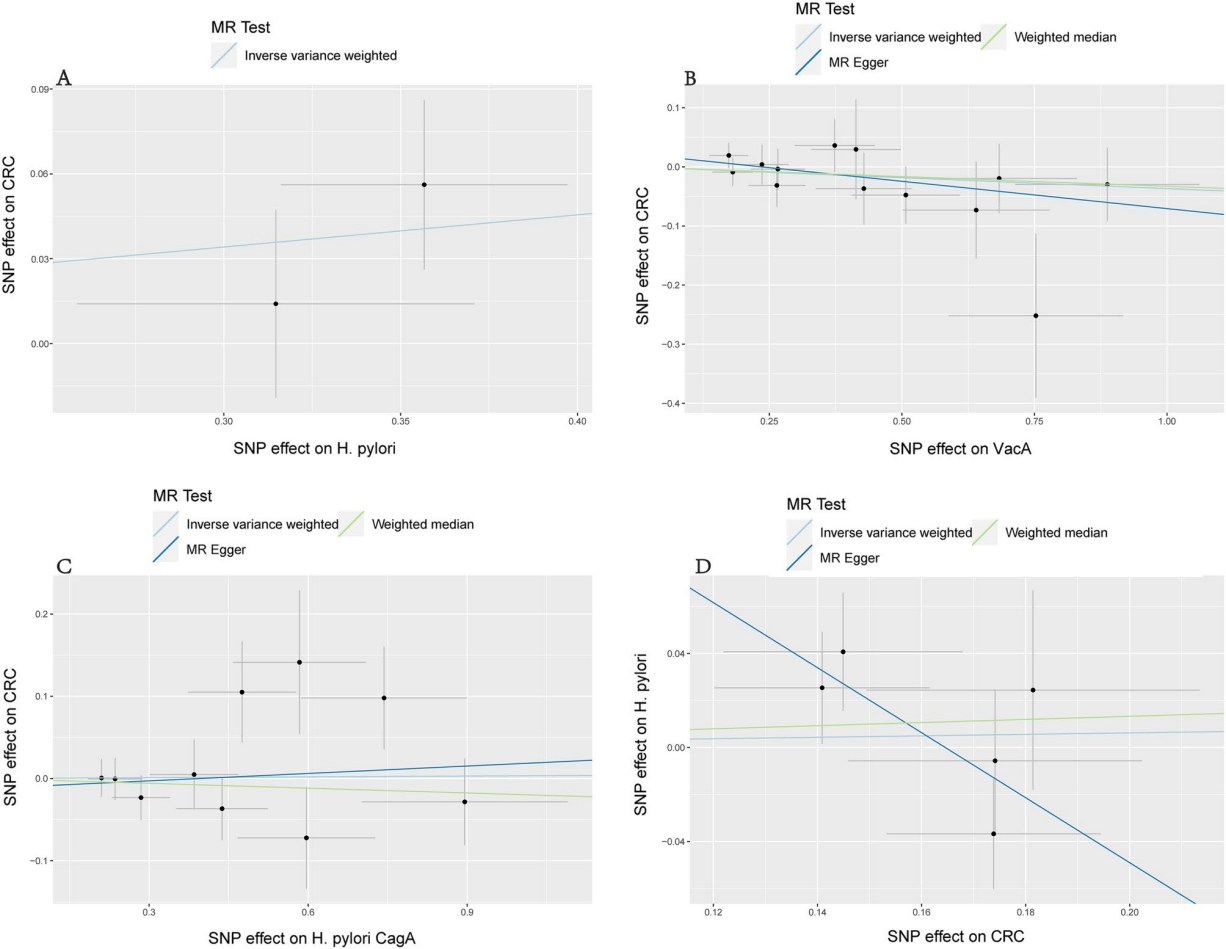
A scatter plot of the causal relationships between *H. pylori* and CRC using different MR methods. (**A**) Causal estimates for *H. pylori* seroprevalence on CRC. (**B**) Causal estimates for VacA on CRC. (**C**) Causal estimates for CagA on CRC. (**D**) Causal estimates for CRC on *H. pylori* infection. The slope of each line corresponds to the causal estimate for each method.

We incorporated 15 independent SNPs with *P* < 5 × 10^–6^ as IV SNPs for anti-*H. pylori* VacA bodies and anti-CagA bodies, respectively. However, two VacA-associated SNPs and five CagA-associated SNPs were not available in the pooled statistics from CRC, as shown in Supplementary Table S3. According to the CC and RC summary statistics provided in Supplementary Table S4, one CagA-associated SNP was missing. There were no F-statistics below 10, and the variation explained by these IVs was close to 22% for VacA and 18.8% for CagA. Our MR analysis has significant power (over 80% to identify an OR of 1.2) to find moderate correlations of VacA and CagA with CRC, CC, and RC; however, it has restricted low power in assessing the effect of *H. pylori* infection on CRC, CC, and RC (66% power to detect an OR of 1.20). The results of MR estimates using several philosophies are presented in Fig. 2. There was no evidence of a causal connection between having a genetic predisposition to *H. pylori* infection and an increased risk of CRC. The primary IVW method showed that genetically predicted *H. pylori* seropositivity in the FinnGen GWAS was not causally associated with CRC [odds ratio (OR) = 1.12, 95% confidence interval (CI): = 0.98–1.27, *P* = 0.08], with CC (OR = 1.00, 95% CI: 0.99–1.00, *P* = 0.81), and with RC (OR = 1.00, 95% CI: 0.99–1.00, *P* = 0.83) in the UK Biobank GWAS. In addition, the MR estimates showed that the causal effect of VacA and CagA on CRC and its subtypes was the same as that of *H. pylori* infection on CRC. In addition, the sensitivity analyses of both MR-Egger and weighted median had comparable conclusions, as shown in Table 3. Scatter plots for effect sizes of SNPs for *H. pylori* infection, anti-VacA antibody, and anti-CagA antibody for CRC, CC, and RC are shown in Fig. 3 and Supplementary Fig. S7. The results of the Cochran Q test revealed no significant heterogeneity, indicating that there was no probable horizontal pleiotropy in any of the estimated s of SNPs in any study. The Cochran Q test showed no heterogeneity. Furthermore, the MR-Egger regression revealed no evidence of directional pleiotropy. The MR-PRESSO results were robust, and no outliers were detected (Supplementary Table S2). A single SNP did not drive the causative estimates of *H. pylori* infection, as shown by a leave-one-out analysis. Supplementary Figs. S2–S6 show the results of leave-one-out analyses and forest and funnel plots.

**Table 3:** MR estimates assessing the bidirectional causal association between *H. pylori* infection and CRC.

Exposure	Outcome	No. of SNPs	R^2^ (%)	F-statistic	IVW		MR-Egger		Weighted median	
					OR (95% CI)	p	OR (95% CI)	p	OR (95% CI)	p
*H. pylori*	CRC	2	7.43	846.96	1.12 (0.98, 1.27)	0.08	–	–	–	–
	CC	2	7.43	846.96	1.00 (0.99, 1.00)	0.81	–	–	–	–
	RC	2	7.43	846.96	1.00 (0.99, 1.00)	0.83	–	–	–	–
VacA	CRC	13	18.82	299.64	0.96 (0.90, 1.02)	0.25	0.91 (0.80, 1.03)	0.16	0.96 (0.89, 1.05)	0.43
	CC	15	21.44	341.41	1.00 (0.99, 1.00)	0.05	1.00 (1.00, 1.00)	0.06	1.00 (1.00, 1.00)	0.01
	RC	15	21.44	341.41	1.00 (1.00, 1.00)	0.05	1.00 (0.99, 1.00)	0.69	1.00 (0.99, 1.00)	0.18
CagA	CRC	10	22.45	225.92	1.00 (0.93, 1.07)	0.92	1.03 (0.88, 1.19)	0.70	0.98 (0.90, 1.06)	0.64
	CC	14	31.82	320.24	1.00 (0.99, 1.00)	0.57	1.00 (0.99, 1.00)	0.70	1.00 (0.99, 1.00)	0.57
	RC	14	31.82	320.24	0.99 (0.99, 1.00)	0.19	0.99 (0.99, 1.00)	0.29	0.99 (0.99, 1.00)	0.35
CRC	*H. pylori*	5	4.68	234.55	1.03 (0.85,1.24)	0.74	0.25 (0.05,1.07)	0.15	1.06 (0.87,1.29)	0.50

*No. of SNPs* number of single-nucleotide polymorphisms, *OR* odds ratio, *CI* confidence interval, *IVW* inverse variance-weighted method, *H. pylori* *Helicobacter pylori*, *VacA* vacuolar cytotoxin A, *CagA* cytotoxin-associated protein A, *CRC* colorectal cancer, *CC* colon cancer, *RC* rectal cancer.

### Causal effect of CRC on *Helicobacter pylori* infection

The IVs of CRC were identified using the FinnGen consortium GWAS. A total of five SNPs, namely rs11213823C, rs16969344G, rs2337113G, rs2735940G, and rs7897408A, were selected. rs11213823C maps to the colorectal cancer-associated 2 gene (COLCA2), which has been identified as being causally associated with the increased risk of CRC^32^. Shorter telomeres^33^ and increased cancer risk^34^ have been associated with the human telomerase reverse transcriptase gene and its related SNP, rs2735940G. Unfortunately, little information and few related publications are currently available on the SNPs rs16969344G, rs2337113G, and rs7897408G.

The IVW method revealed no evidence for a correlation between genetically predicted CRC and *H. pylori* infection. (OR = 1.03, 95% CI: 0.85–1.24, *P* = 0.74). The weighted median approach (OR = 1.06, 95% CI: 0.87–1.29, *P* = 0.50) and the MR-Egger method (OR = 0.25, 95% CI = 0.05–1.07, *P* = 0.15) generated identical results, as shown in Fig. 2. There was no heterogeneity, no directed polymorphism, and no outlier detected, as shown in Supplementary Table S2.

## Discussions

Using four genetic data pools, our study did not directly compare *H. pylori* serology and colorectal cancer (CRC). Instead, we employed a Mendelian randomization approach using potential single nucleotide polymorphisms (SNPs) associated with *H. pylori* infection as instrumental variables to estimate the possible causality between *H. pylori* and CRC. Our results did not show any clear evidence of a causal effect of genetic predisposition to *H. pylori* infection on CRC risk. Multiple MR sensitivity analysis methods showed consistent results.

Regarding the association between *H. pylori* infection and CRC, we acknowledge that *H. pylori* infection is influenced by hygiene standards and family history. While our study focused on the potential genetic links between *H. pylori* and CRC, we cited previous research, such as the study by Mayerle et al.^23^, to provide additional context. We acknowledge that further replication and validation of the identified SNPs as surrogate biomarkers for predicting *H. pylori* infection are necessary. However, our study aimed to explore the potential genetic factors underlying the association, which can contribute to a better understanding of the biology of *H. pylori*-related CRC^35^.

To our knowledge, there is no inconsistent evidence on the association of *H. pylori* infection with an increased risk of CRC. A study on a large consortium of cohorts, which included ten prospective cohorts of American populations, conducted by Butt et al.^8^ in 2019 revealed that *H. pylori* VacA-specific seropositivity was associated with an increased risk of CRC (OR, 1.11; 95% CI, 1.01–1.22). In another study involving the same cohorts performed in 2020, Epplein et al.^36^ analyzed *H. pylori* VacA toxin levels and performed the *Streptococcus gallolyticus* pilus protein assay and found a significant association between *H. pylori* and CRC risk. A meta-analysis of 27 studies by Yang et al.^37^ conducted in 2019 and another meta-analysis of 48 studies by Choi et al.^38^ conducted in 2020 report similar conclusions. Additionally, the findings of another cohort study conducted by Wang et al.^39^ in 2020 in China support that *H. pylori* infection increases the risk of colorectal polyps and malignancy.

Nevertheless, a multi case–control research carried out by Fernández et al.^40^ in Spain in 2017 found that neither *H. pylori* seropositivity nor CagA and VacA were associated with a higher risk of CRC (OR, 0.91; 95% CI: 0.71–1.16). Regarding anti-CagA antibodies, their presence in H. pylori infection is not universal. Different studies have reported varying rates of anti-CagA antibody development. Nonetheless, our study did not solely rely on anti-CagA antibodies for the analysis but considered a broader perspective by investigating potential genetic associations. Moreover, in Blase et al.’s^41^ nested case–control study that involved individuals of Caucasian, they reported that *H. pylori* infection was not associated with CRC. A cross-sectional study by Boyuk et al.^11^ in 2019 also reported similar conclusions. In 2014, Guo et al.^42^ performed a meta-analysis of studies on the East Asian population to further investigate the association between *H. pylori* infection and colorectal neoplasm; their results showed that *H. pylori* infection had no apparent association with colorectal neoplasm. The studies of Patel et al.^10^ on the Hispanic population in the United States, Limburg et al.^43^ on Finnish population, and Machida-Montani et al.^44^ on Japanese population all support the absence of a correlation between *H. pylori* infection and colorectal malignancies.

According to the two large meta-analyses, one by Hooi et al.^45^ and the other by Ren et al.^46^, the prevalence of *H. pylori* infection is roughly 79.1% in Africa, 54.7% in Asia, 47% in Europe, 44.2% in the mainland of China, and 37.1% in North America. In a subgroup analysis of the cohort study conducted by Butt et al.^8^, they reported that *H. pylori* infection raises the risk of CRC in African Americans. Another study by Blase et al.^41^ refuted the notion that *H. pylori* infection was associated with a higher risk of CRC in elderly Caucasian population.

There are a number of possible explanations for the controversy surrounding *H. pylori* infection and the risk of CRC. First, there were no prospective, randomized, or blinded methods in any of these epidemiologic observational studies. The discrepancies in findings are most likely attributable to biases associated with improper confounding control, selection bias, or reverse causality. Second, different studies have used different diagnostic strategies for CRC and *H. pylori* infections. Based on the global guidelines of the World Gastroenterology Organization (WGO)^47^, the urine breath test is the most recommended non-invasive test to check for *H. pylori* infection. However, some studies measured *H. pylori* infection using serum or IgG antibodies to *H. pylori* or *H. pylori* DNA sequences, and therefore, the criteria for the diagnosis of *H. pylori* infection were inconsistent across studies^8,12,48,49^. Third, although biopsy is the standard method for diagnosing CRC, some studies obtained specimens via surgery, and these surgical specimens revealed inconsistent pathological classification. Fourth, these differences could be attributed to differences in the age, sex, geographic region, ethnicity, dietary habits, socioeconomic status, anti-*H. pylori* IgG titers, and anti-*H. pylori* antibody types and levels.

We acknowledge that CRC is a multifactorial disease influenced by various non-hereditary factors. However, genetic susceptibility can still play a role in disease development, including gene-environment interactions. By exploring the potential genetic links between H. pylori and CRC, our study aimed to contribute to the understanding of the complex etiology of CRC. As drawing conclusions from observational studies to avoid confounding risk factors has been difficult, it is important to investigate the mechanisms underlying the infection caused by *H. pylori* that lead to CRC. With MR methods, causality can be revealed reliably and without bias because MR is a natural randomized controlled trial performed during fertilization and has similar randomized controlled trials study design. Based on the rigorous assumptions regarding the relationship between the genetic score for exposure and the outcome, as underlying the principles of Mendelian Randomization, there is no compelling evidence suggesting a causal association between infection and colorectal cancer. Our study is the first attempt to elucidate the connection between *H. pylori* infection and CRC from the perspective of genetic variation.

The findings of the GWAS investigation yielded only two SNPs (rs10004195 and rs368433) that were significantly and independently linked to *H. pylori* infection. The SNP rs10004195 was found at the toll-like receptor (TLR) locus on chromosome 4p14, and the SNP rs368433 was found at the FCGR2A locus on chromosome 1q23. TLRs, the pattern recognition receptors with the most recognizable features, are critical for both innate and adaptive immune responses^50^. Tang et al.^51^ published their findings on TLR10 (rs10004195) and *H. pylori* infection in a Chinese population in 2015; they discovered that TLR10 (rs10004195) had a protective effect against *H. pylori* infection (OR = 0.83; 95% CI: 0.72–0.96). Another study conducted by EI-Omar et al.^52^ reported that low expression of the TLR rs10004195 was associated with increased protection against *H. pylori* infection, whereas high expression of the TLR rs10004195 was associated with *H. pylori* seropositivity. Two meta-analyses performed respectively by Karpiski et al.^53^ and Moradi-Marjaneh et al.^54^ revealed that gastrointestinal microorganisms play a significant role in colorectal carcinogenesis. Notably, most ongoing studies are at the stage of animal experiments^55^, and the potential association between TLRs and CRC signaling pathways still needs to be further investigated and confirmed in clinical trials. The SNP rs368433 is associated with the FCGR2A gene. Zhang et al.^56^ found that patients treated with cetuximab for CRC had a poorer outcome when they had the FCGR2A genotype. Geva et al.^57^ reported that no differences were found between FCGR polymorphisms when treated with cetuximab.

There have been some studies on virulence factors of *H. pylori* infection and their impact on the host immune response and physiology of gastric disease^58,59^. However, to our knowledge, no precise information on signaling pathways associating *H. pylori* infection with CRC pathogenesis is currently available, and the overall information on the contribution of *H. pylori*-related extraintestinal disorders to CRC is too limited to be fully understood. Thus far, there has been no report identifying a direct molecular signaling pathway leading to CRC through SNPs associated with *H. pylori* infection, and there is no evidence for the presence of *H. pylori* or corresponding proteins in CRC tissues. Further studies are warranted to confirm the presence of *H. pylori* in the colorectal epithelium and its potential direct effects in causing CRC^59^. Our data suggest no direct causative link between *H. pylori* infection and CRC incidence, and bidirectional MR analysis was performed to clarify the causality. Our study has several notable strengths. First, we could simulate randomized controlled trials in observational settings using the MR design. Although randomized controlled trials are widely considered the most reliable approach to identify the presence of a causal relationship, it is not always possible to carry one out due to its high cost. However, since SNPs are randomly allocated at conception, MR studies can successfully correct for this potential confounding bias. Unlike the approaches used in other observational studies, MR offers the advantage of being able to address the reverse causality problem. Second, our study results may have implications for healthcare policies regarding *H. pylori* infection and CRC. Given the high incidence of *H. pylori* infection in the general population, establishing causation between *H. pylori* infection and CRC can affect public health strategies for early prevention and appropriate intervention. Our results indicate that people with genetic predisposition to *H. pylori* infection may not benefit from CRC screening.

In summary, while we acknowledge the limitations and complexities of studying the association between *H. pylori* and CRC, we believe that our study provides valuable insights by employing a Mendelian randomization approach and considering potential genetic factors.

## Conclusions

This MR study was aimed at investigating the causality of *H. pylori* infection with CRC. The findings do not lend credence to the notion that a higher seroprevalence of *H. pylori* infection is associated with a higher risk of CRC caused by genetic variation.

### Supplementary Information

Supplementary Information.

## Data Availability

The GWAS data related to CRC were sourced from the FinnGen website at https://www.finngen.fi/en. The summary-level data for CC and RC were obtained from the Neale Lab UK Biobank portal, available at http://www.nealelab.is/uk-biobank/. Additionally, the consolidated GWAS data can be found at the MRC IEU OpenGWAS database, located at https://gwas.mrcieu.ac.uk/datasets.

## Abbreviations

*H. pylori*: *Helicobacter pylori*
CRC: Colorectal cancer
CC: Colon cancer
RC: Rectal cancer
SNPs: Single-nucleotide polymorphisms
IVs: Instrumental variants
EBI: European Bioinformatics Institute
IVW: Inverse variance weighted
MR: Mendelian randomization
MR-PRESSO: MR Pleiotropy Residual Sum and Outlier
nSNPs: The number of SNPs used in the analysis
OR: Odds ratio
VacA: Vacuolar cytotoxin A
CagA: Cytotoxin-associated protein A
CI: Confidence interval
