# Supplementary material for: No evident causal association between Helicobacter pylori infection and colorectal cancer: a bidirectional mendelian randomization study
Source: Sci Rep. 2023 Oct 29;13:18544. doi: 10.1038/s41598-023-45545-x (PMC10613620; doi:10.1038/s41598-023-45545-x)
Supplement: Supplementary file 1 — Supplementary Information. [file 41598_2023_45545_MOESM1_ESM.pdf]

Supplementary Materials

Table S1. Instrumental variants of CRC and F statistic

SNPs	Beta	SE	EAF	*P* value	Gene	F statistic
rs11213823C	-0.14493	0.022955	0.739396	2.72E-10	COLCA2	2522.93
rs16969344G	0.181418	0.032016	0.107535	1.46E-08	-	1965.42
rs2337113G	-0.17385	0.020569	0.506603	2.86E-17	-	4742.66
rs2735940G	0.140898	0.02074	0.530075	1.09E-11	TERT	3088.12
rs7897408A	0.174125	0.028242	0.143031	7.03E-10	-	2315.04

"-" represents not reported.

Abbreviations: SNPs, single-nucleotide polymorphism; EAF, effect allele frequency; CRC, colorectal cancer

Table S2. MR estimates of assessing the bidirectional causal association between *H. pylori* and CRC

Exposure	Outcome	No of SNPs	F-statistic	IVW		MR-Egger regression		MR-PRESSO
				Q statistic	Q_pval	Intercept	P_inter	P_ global heterogeneity
H. pylori	CRC	2	846.96	0.69	0.40	-	-	-
	CC	2	846.96	4.69	0.03	-	-	-
	RC	2	846.96	0.48	0.48	-	-	-
VacA	CRC	13	299.64	6.14	0.90	0.017	0.41	0.88
	CC	15	341.41	15.69	0.33	-0.001	0.23	0.35
	RC	15	341.41	11.71	0.63	0.001	0.55	0.62
CagA	CRC	10	225.92	12.54	0.18	-0.153	0.63	0.29
	CC	14	320.24	5.69	0.95	-0.001	0.84	0.95
	RC	14	320.24	17.91	0.16	0.001	0.52	0.18
CRC	*H. pylori*	5	234.55	6.42	0.16	0.227	0.15	0.20

P_inter: *P* value for intercept test of multivariable MR-Egger

P_ global heterogeneity: p value for global heterogeneity test of MR-PRESSO

Abbreviations: No of SNPs, number of single-nucleotide polymorphisms; IVW, inverse variance-weighted average method; *H. pylori*, *Helicobacter pylori*; VacA, Vacuolar cytotoxin A; CagA, Cytotoxin-associated protein A; CRC, colorectal cancer

Table S3 Detailed information of instrumental variables used in the Mendelian randomization analysis of VacA on CRC, CC, and RC

			associated with VacA					associated with CRC			associated with CC			associated with RC		
SNP	EA/OA	eaf	beta	se	pval.exposure	Rsquare	Fvalue	beta	se	pval.exposure	beta	se	pval.exposure	beta.outcome	se.outcome	pval.outcor
rs10246445	C/A	0.028197	-0.488144	0.106857	4.91972E-06	0.013058914	20.7605455	#N/A	#N/A	#N/A	0.00065069	0.00043842	0.137761	-7.1882E-05	0.000207975	0.729621
rs11044935	T/A	0.039367	0.428278	0.0905091	2.22454E-06	0.013873031	22.0730047	-0.0367792	0.0605418	0.543518	0.00042318	0.00033552	0.207217	-6.2068E-05	0.000159163	0.696561
rs113063793	T/A	0.056242	0.373402	0.075648	7.97223E-07	0.014801464	23.5724033	0.0360762	0.0450013	0.422744	0.00043758	0.00028762	0.128166	-1.0441E-05	0.00013644	0.939004
rs113845906	A/G	0.045066	0.413478	0.0845187	9.97401E-07	0.014714895	23.4324773	0.0295301	0.0844661	0.726631	0.00032506	0.0003085	0.292031	0.00042832	0.000146344	0.003425
rs117077218	T/C	0.016656	0.640222	0.138755	3.94903E-06	0.013426641	21.3530997	-0.0732318	0.0818982	0.371225	0.00046389	0.00056173	0.408909	0.00021268	0.000266472	0.424789
rs133537	C/T	0.615079	-0.173609	0.0367088	2.25237E-06	0.014271742	22.7165674	-0.0192828	0.0210663	0.360014	3.7906E-05	0.00014166	0.789013	-3.7739E-05	6.71984E-05	0.574382
rs148556020	A/T	0.016883	0.63085	0.137457	4.44427E-06	0.013211041	21.0056294	#N/A	#N/A	#N/A	0.00099026	0.0005574	0.0756404	0.00043679	0.000264418	0.09856
rs1530121	C/T	0.86313	-0.266111	0.0511414	1.95637E-07	0.016731702	26.6987565	0.00389742	0.0347269	0.910641	-0.00025028	0.00019908	0.208688	-5.979E-05	9.44399E-05	0.526666
rs372744619	G/A	0.010519	0.887411	0.173802	3.29276E-07	0.016393117	26.1494711	-0.029835	0.0620667	0.630735	0.00093456	0.00057581	0.104581	-0.00018947	0.000273151	0.487917
rs59104649	T/A	0.014016	0.68347	0.147207	3.43534E-06	0.012911089	20.5224656	-0.0196325	0.0586802	0.73795	-0.00031551	0.00066074	0.632996	4.4923E-05	0.000313437	0.886036
rs7019543	T/G	0.320078	0.18107	0.0380994	2.00845E-06	0.014270458	22.7144951	-0.00884358	0.0235195	0.706908	-5.0233E-05	0.00014468	0.728448	5.1643E-05	0.000068634	0.451788
rs72645538	G/A	0.011992	0.752792	0.164377	4.65715E-06	0.013428641	21.3563243	-0.251824	0.138988	0.0700116	0.00069367	0.00050981	0.173621	-9.314E-05	0.00024184	0.70014
rs73500239	C/G	0.144805	0.236088	0.0500401	2.38199E-06	0.013804686	21.9627411	0.00393427	0.0340207	0.907935	0.00017035	0.00019462	0.381413	8.1317E-05	9.23206E-05	0.378423
rs77497849	G/A	0.119223	0.264227	0.0538191	9.12935E-07	0.01466258	23.3479289	-0.031408	0.0359599	0.382435	-0.00020617	0.00020678	0.318735	8.1094E-06	9.80909E-05	0.934112
rs9606224	T/C	0.029841	0.507461	0.10223	6.90876E-07	0.01491048	23.748647	-0.047712	0.0482359	0.322595	-0.00028191	0.00038259	0.461207	0.00015503	0.00018149	0.392993

Abbreviations: VacA, vacuolar cytotoxin A; CRC, colorectal cancer; CC, colon cancer; RC, rectal cancer; EA/OA, effect allele/other allele

Table S4 Detailed information of instrumental variables used in the Mendelian randomization analysis of CagA on CRC, CC, and RC

		associated with CagA						associated with CRC			associated with CC			associated with CC		
SNP	EA/OA	eaf	beta	se	pval	Rsquare	Fvalue	beta	se	pval	beta	se	pval	beta	se	pval
rs117537486	C/G	0.03072	0.596789	0.129736	4.22E-06	0.02121	21.3013	-0.0721833	0.0619749	0.244132	0.000276187	0.000395	0.483952	-0.0001	0.000187	0.576066
rs117827497	G/A	0.030462	-0.58373	0.125147	3.10E-06	0.020127	20.19122	-0.141312	0.0873738	0.105807	0.000114445	0.000419	0.784634	0.000591	0.000199	0.002911
rs118006294	C/T	0.050367	-0.4753	0.101815	3.04E-06	0.02161	21.71201	-0.104967	0.061496	0.0878416	-0.000294033	0.000315	0.351352	0.000161	0.00015	0.280808
rs11858369	G/A	0.068381	0.437721	0.086498	4.18E-07	0.024412	24.59719	-0.0366689	0.0386895	0.343246	-9.56601E-05	0.000274	0.727009	-3.5E-05	0.00013	0.785753
rs149747348	C/G	0.013186	-0.89541	0.194003	3.92E-06	0.020865	20.94741	0.0283986	0.0525974	0.589249	0.000263343	0.000626	0.67383	0.000154	0.000297	0.604828
rs3998182	C/T	0.27555	0.284762	0.054805	2.04E-07	0.032374	32.88888	-0.0232392	0.0272545	0.39384	3.21089E-05	0.000161	0.841605	-0.00011	7.62E-05	0.160046
rs4268452	T/C	0.071203	-0.38498	0.084033	4.62E-06	0.019603	19.65476	-0.00483088	0.0424041	0.909297	-9.52231E-05	0.000267	0.721841	5.24E-06	0.000127	0.967039
rs6530847	A/T	0.391211	-0.21027	0.045273	3.41E-06	0.021059	21.14673	-0.000666945	0.0231241	0.976991	0.00001856	0.00014	0.894865	-3.8E-05	6.66E-05	0.564062
rs75170215	C/T	0.02057	0.743256	0.156416	2.02E-06	0.022259	22.37919	0.0978776	0.0625676	0.117735	0.000347531	0.000481	0.469642	0.000367	0.000228	0.107309
rs75740599	A/G	0.252886	0.235842	0.051296	4.27E-06	0.021018	21.10389	-0.000490338	0.0256342	0.984739	3.28916E-05	0.000159	0.835708	-0.00013	7.52E-05	0.092961

Abbreviations: CagA, Cytotoxin-associated protein A; CRC, colorectal cancer; CC, colon cancer; RC, rectal cancer; EA/OA, effect allele/other allele

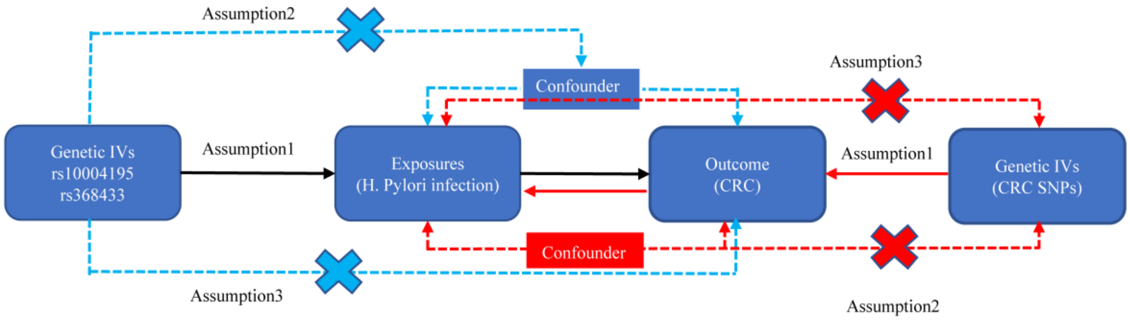

Figure S1. The overview of the bidirectional MR study on the causal relationship between *H. pylori* infection and CRC

Abbreviations: SNPs, single-nucleotide polymorphisms; IVs, instrumental variants; *H. pylori*, *Helicobacter pylori*; CRC, colorectal cancer

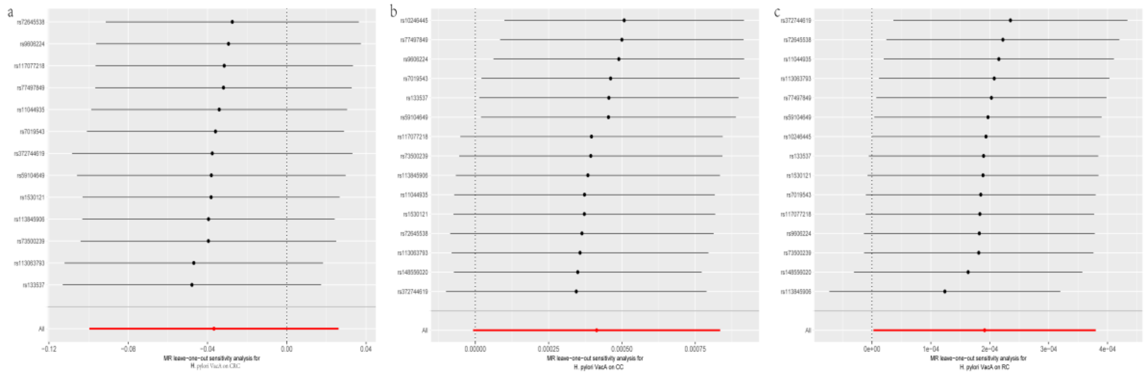

Figure S2. The leave-one-out plot of the causal relationships of VacA with CRC, CC, and RC. (a) MR estimates for VacA on CRC. (b) MR estimates for VacA on CC. (c) MR estimates for VacA on RC. The leave-one-out plot visualized how the causal estimates (point with horizontal line) of VacA with CRC, CC, and RC were influenced by the removal of a single variant

Abbreviations: VacA, vacuolating cytotoxin gene A; CRC, colorectal cancer; CC, colon cancer; RC, rectal cancer

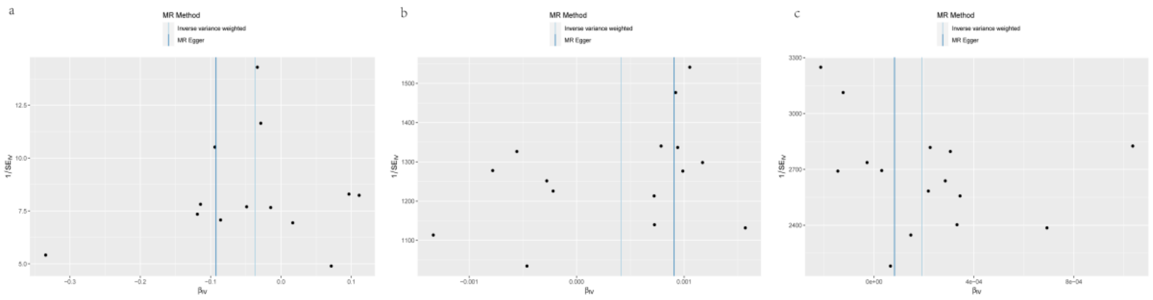

Figure S3. Funnel plot of the causal relationships of VacA with CRC, CC, and RC. (a) MR estimates for VacA on CRC. (b) MR estimates for VacA on CC. (c) MR estimates for VacA on RC. The funnel plot illustrated the overall symmetry of causal estimates across all instrumental variables.

Abbreviations: VacA, vacuolating cytotoxin gene A; CRC, colorectal cancer; CC, colon cancer; RC, rectal cancer

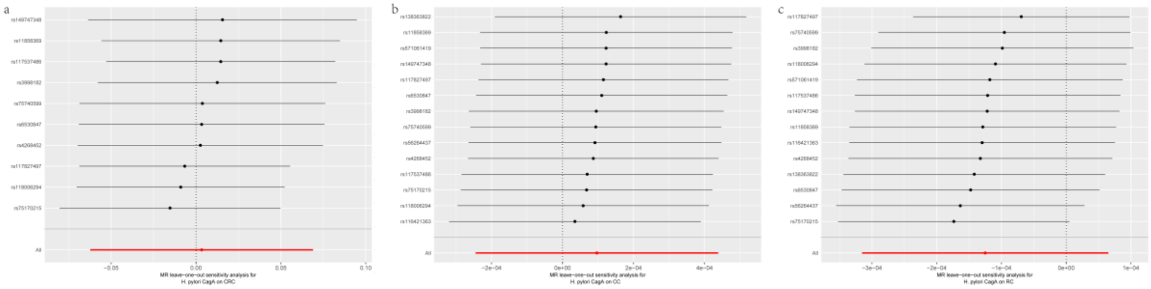

Figure S4. The leave-one-out plot of the causal relationships of CagA with CRC, CC, and RC. (a) MR estimates for CagA on CRC. (b) MR estimates for CagA on CC. (c) MR estimates for VacA on RC. The leave-one-out plot visualized how the causal estimates (point with horizontal line) of CagA with CRC, CC, and RC were influenced by the removal of a single variant.

Abbreviations: CagA, cytotoxin-associated gene A; CRC, colorectal cancer; CC, colon cancer; RC, rectal cancer

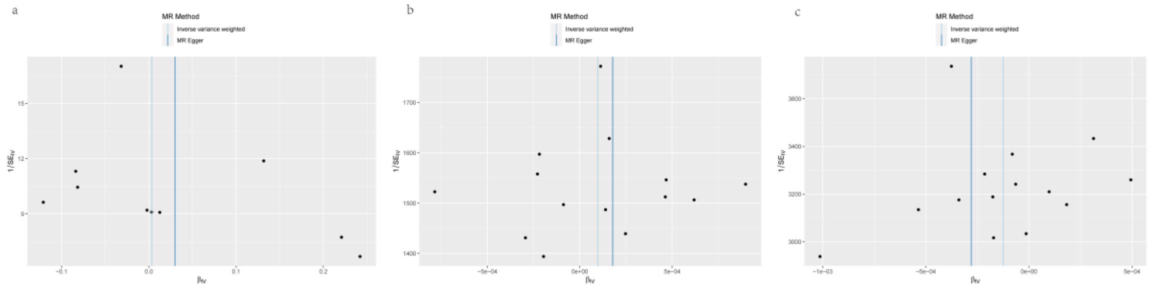

Figure S5. Funnel plot of the causal relationships of CagA with CRC, CC, and RC. (a) MR estimates for CagA on CRC. (b) MR estimates for CagA on CC. (c) MR estimates for CagA on RC. The funnel plot illustrated the overall symmetry of causal estimates across all instrumental variables.

Abbreviations: CagA, cytotoxin-associated gene A; CRC, colorectal cancer; CC, colon cancer; RC, rectal cancer

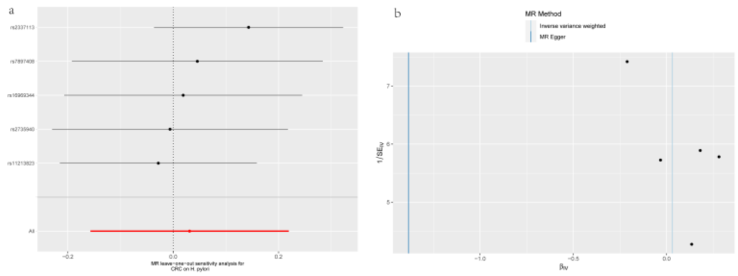

Figure S6. The leave-one-out plot of the causal relationships between CRC and *H. pylori* infection. (a) MR estimates for CRC on *H. pylori* infection. The leave-one-out plot visualized how the causal estimates (point with horizontal line) between CRC were influenced by the removal of a single variant. (b) Funnel plot of the causal relationships between CRC and *H. pylori* infection. The funnel plot illustrated the overall symmetry of causal estimates across all instrumental variables.

Abbreviations: *H. pylori*, *Helicobacter pylori*; CRC, colorectal cancer

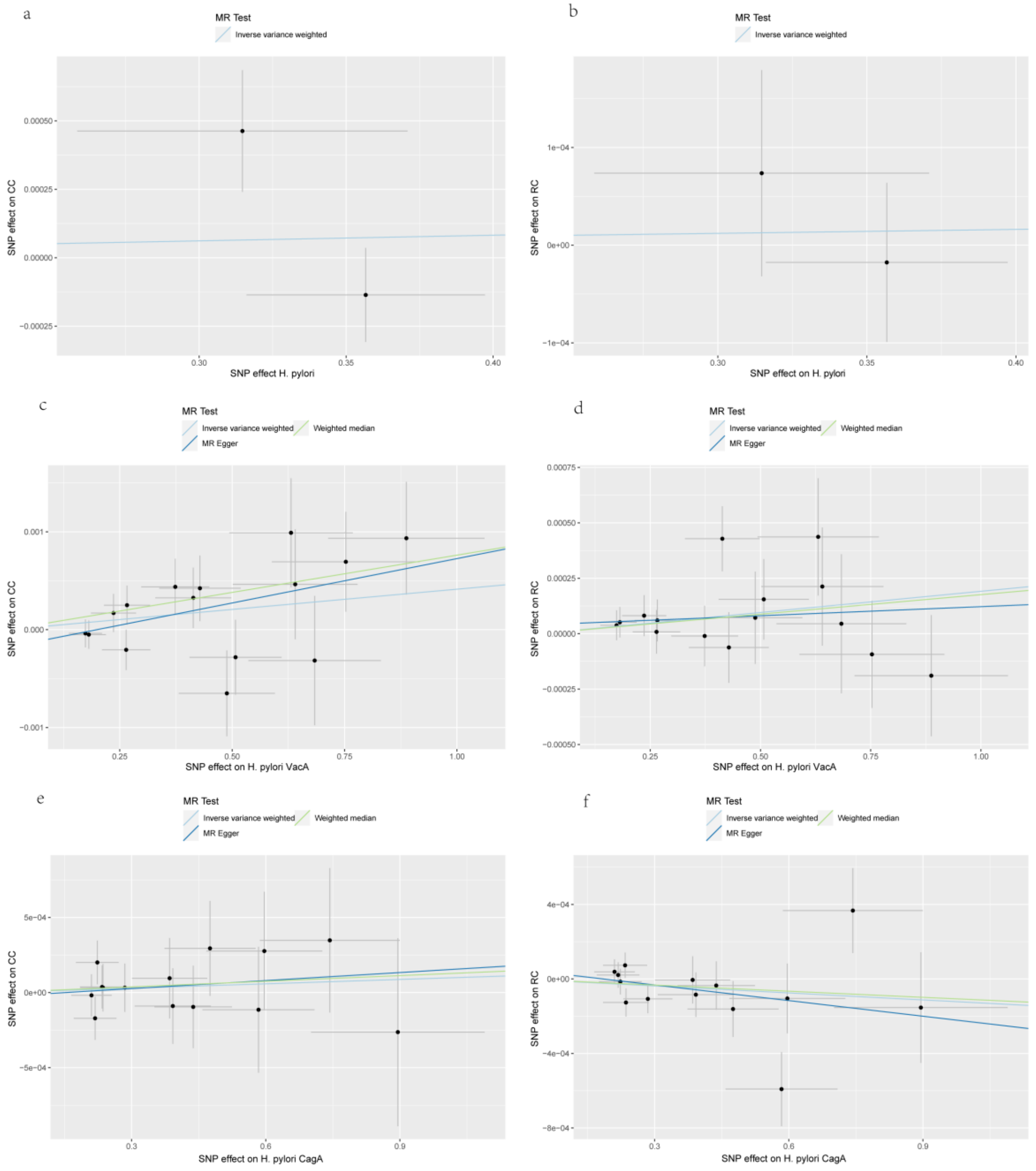

Figure S7. Scatter plot of the causal relationships of *H. pylori*, *H. pylori* VacA, and *H. pylori* CagA with SNP effect on CC and RC using different MR methods. (a) Causal estimates for *H. pylori* on CC. (b) Causal estimates for *H. pylori* on RC. (c) Causal estimates for CavA on CC. (d) Causal estimates for VacA on RC. (e) Causal estimates for CagA on CC. (f) Causal estimates for CagA on RC. The slope of each line corresponds to the causal estimate for each method. The

effect of an individual SNP on the outcome (point and vertical line) against its effect on the exposure (point and horizontal line) was delineated in the background.

Abbreviations: *H. pylori*, *Helicobacter pylori*; SNP, single-nucleotide polymorphism; VacA, vacuolating cytotoxin gene A; CagA, cytotoxin-associated gene A; CC, colon cancer; RC, rectal cancer
